# Lupus miliaris disseminatus faciei with madarosis: A novel presentation

**DOI:** 10.1097/MD.0000000000046552

**Published:** 2025-12-12

**Authors:** Yuanting Su, Wei Cui

**Affiliations:** a Department of Dermatology, The Second People’s Hospital of Hefei, Hefei, Anhui, China.

**Keywords:** lupus miliaris disseminatus faciei, madarosis, scar, pathogenesis

## Abstract

**Rationale:**

Lupus miliaris disseminatus faciei (LMDF) is a chronic, self-limiting inflammatory skin disease of unknown etiology that can lead to disfiguring scars.

**Patient concerns:**

A 51-year-old male presented with a 1-month history of facial papules and eyebrow exfoliation.

**Diagnoses:**

Skin biopsy confirmed that this case was LMDF.

**Interventions:**

After the diagnosis was confirmed, the patient was treated with oral doxycycline (100 mg) and prednisone (12 mg) once daily.

**Outcomes:**

Follow-up at 1 month showed significant improvement in facial rash and regrowth of both eyebrows.

**Lessons:**

This report describes an atypical case of LMDF complicated by eyebrow loss, providing insights into the rarity and pathogenesis of this condition.

## 1. Case report

**Figure 1.**
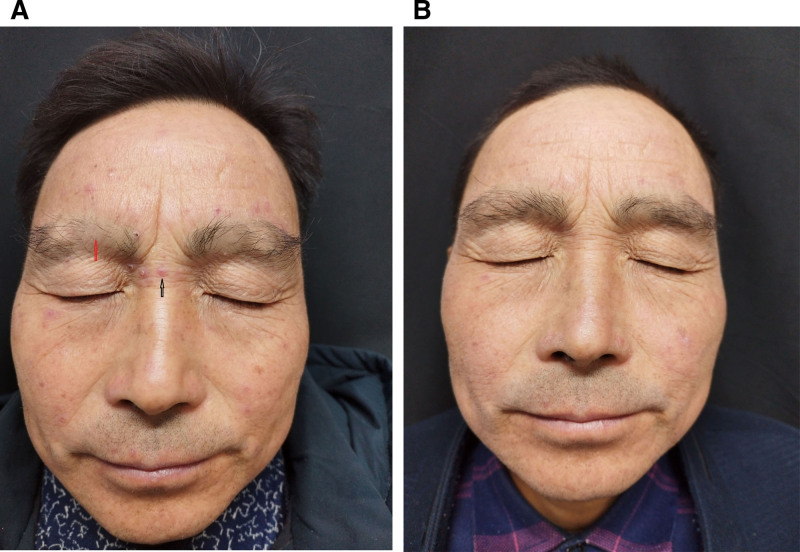
Pretreatment facial manifestations. (A) The lesions were scattered reddish papules(red arrow), measuring 2 to 4 mm in diameter with smooth surfaces and firm consistency on palpation, around the bilateral eyelids and forehead, and localized loss of bilateral eyebrows (black arrow) was observed; (B) nodules and papules subsided with local residual pigmentation after 4 wk of treatment, and growth in both eyebrows.

A 51-year-old male presented to our department on September 3, 2024, with a 1-month history of progressive facial papules and eyebrow loss. Specifically, he had developed an increasing number of pink papules around the bilateral orbits and forehead, accompanied by concurrent eyebrow loss, in the absence of an obvious cause, pruritus, or pain. He denied any prior medical conditions, history of tuberculosis, or relevant family history of dermatological disorders. General physical examination revealed no abnormalities. The patient had not received any relevant treatment for the current condition prior to consultation. Dermatological examination revealed symmetrically distributed, discrete reddish-brown papules involving the forehead, upper/lower eyelids, and malar regions; these papules were most densely clustered in the periorbital area, measuring 2 to 4 mm in diameter with smooth surfaces, and palpation revealed a firm texture. Notably, symmetric eyebrow alopecia (loss of the middle-inner 1/3 of the eyebrows), and the grid method (4 × 4 mm², 20 units) showed 10 affected units (≈50% alopecia rate) (Fig. 1A).

**Figure 2.**
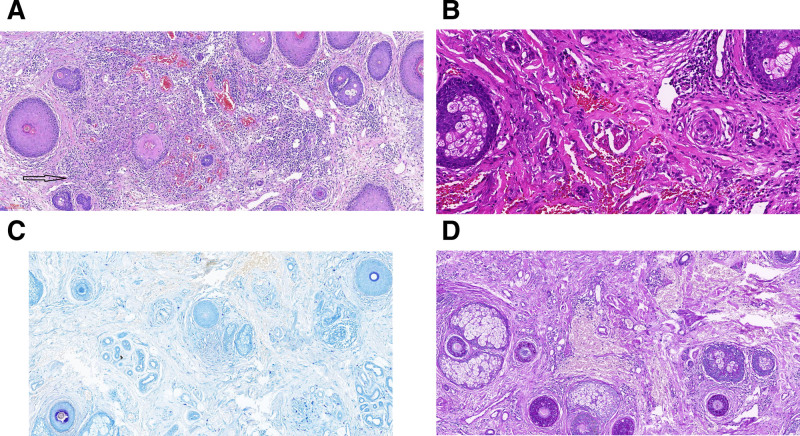
HE staining showed central caseous necrosis surrounded by a dense infiltrate of inflammatory cells, along with collagen fiber degeneration (black arrow) and vascular thrombosis. (A, 40×/B, 100×) and acid-fast staining (C) and PAS staining (D) were negative. HE = hematoxylin-eosin, PAS = periodic acid-Schiff.

**Figure 3.**
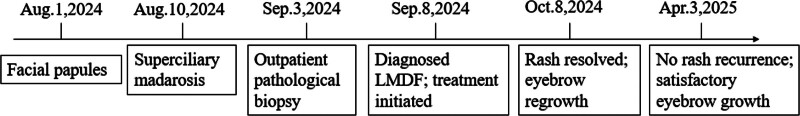
The timeline of the patient’s clinical evolution.

Laboratory testing showed normal results for the antinuclear antibody spectrum, tuberculin skin test, and chest computed tomography scan. A histopathological examination was performed to confirm the diagnosis. Histopathological examination demonstrated central caseous necrosis surrounded by a dense infiltration of inflammatory cells, including epithelioid cells and lymphocytes, along with collagen fiber degeneration and vascular thrombosis (Fig. 2A and B). Additionally, acid-fast staining (Fig. 2C) and periodic acid-Schiff staining were negative (Fig. 2D). Based on these findings, a diagnosis of lupus miliaris disseminatus faciei (LMDF) was confirmed. The patient was initiated on oral doxycycline (100 mg) and prednisone (12 mg), each administered once daily. At the 1-month follow-up visit (October 8, 2024), significant resolution of the facial rash and regrowth of the bilateral eyebrows were observed (Fig. 1B). A telephone follow-up was conducted 6 months later (until April 4, 2025), and no recurrence of the rash was observed (Fig. 3).

## 2. Discussion

LMDF is a chronic, inflammatory, granulomatous dermatosis of unknown etiology. It is widely regarded as unrelated to tuberculosis infection. LMDF predominantly affects young to middle-aged males. Clinically, it is characterized by pale-red to reddish-brown papules or nodules with smooth surfaces and firm textures, notably; these lesions exhibit an “apple sauce” hue when pressed with a glass slide. The size of the lesions typically ranges from that of a millet grain to a mung bean.^[1]^ The disease course is usually protracted, spanning several months to years; while some lesions may resolve spontaneously, this resolution is often accompanied by the formation of atrophic scars.^[2]^ Importantly, LMDF is generally not associated with concurrent comorbidities, and relevant reports on comorbidities linked to LMDF remain scarce.^[3]^ To the best of our knowledge, this is the first reported case of LMDF presenting with facial pink papules and bilateral eyebrow alopecia, which achieved significant clinical improvement following pharmacotherapy.

Unlike typical LMDF cases, this patient presented with concurrent eyebrow alopecia – a novel clinical manifestation that warrants further investigation. As a speculative analysis based on current research progress, we propose that the pathogenesis of LMDF may involve an aberrant cellular immune response to *Propionibacterium acnes (P. acnes*) following the destruction of folliculosebaceous units, accompanied by abnormally elevated levels of transforming growth factor-β1 (TGF-β1) within lesional skin.^[4]^ Notably, accumulating evidence has also linked *P. acnes* to alopecia: this bacterium can elicit perifollicular inflammation and impair hair follicle regeneration.^[5]^ Furthermore, TGF-β1 has been demonstrated to accelerate the transition of hair follicles from the anagen (growth) phase to the telogen (resting) phase in the hair growth cycle, thereby inhibiting hair follicle proliferation and migration.^[6]^

In the context of the current patient, we propose that eyebrow alopecia may be linked to a local *P. acnes*-induced immune response and elevated TGF-β1. This pathway could disrupt normal hair follicle homeostasis, ultimately leading to eyebrow alopecia in this LMDF case. However, this study has not yet obtained direct histopathological evidence to support this hypothesis. Further well-designed clinical studies and mechanistic investigations are necessary to validate this proposed correlation and clarify the underlying pathophysiological link between LMDF and associated eyebrow alopecia.

There is currently a scarcity of studies and publications on the therapy of LMDF. The available treatment choices include tetracyclines, isotretinoin, dapsone, and corticosteroids. Commencing treatment at the early stage reduces the duration of treatment and minimizes the incidence of scars. Nevertheless, the efficacy of these therapies remains a subject of discussion, and there is presently no standardized therapeutic protocol. Notably, doxycycline can inhibit the activation of neutrophils and macrophages as well as the synthesis of inflammatory factors. When used in combination with prednisone, it can enhance the anti-inflammatory effect, while reducing the dosage of prednisone and minimizing prednisone-related side effects. Compared with isotretinoin or dapsone, the combined administration of prednisone and doxycycline offers a more rapid onset of efficacy within 1 to 2 weeks. Nasimi et al analyzed 70 LMDF cases and found 75.7% of granulomas were associated with pilosebaceous units, confirming the need to target follicular inflammation – aligning with doxycycline’s anti-inflammatory role in this region and prednisone’s ability to suppress granulomas.^[3]^ Wang et al reported that 88.63% of 109 LMDF patients developed atrophic scars with delayed treatment, which underscores the value of our regimen’s 1-month lesion resolution for scar prevention.^[1]^ Matsuno et al further validated this combination: a patient unresponsive to tetracycline monotherapy achieved 1-year remission with “minocycline + low-dose prednisone,” reinforcing our choice of dual therapy over monotherapy.^[7]^ Meanwhile, Kwon et al highlighted limitations of alternatives (e.g., minocycline, isotretinoin, cyclosporine) in a refractory case – including inefficacy or side effects – emphasizing our regimen’s better balance of efficacy and tolerability for first-line use.^[8]^ Furthermore, this combination regimen is suitable for women of childbearing age and exerts a milder impact on hepatic and renal function. Given the observed clinical benefits of this combination regimen relative to monotherapy, we opted to initiate systemic doxycycline plus prednisone in the patient described in this case. The selection of the combination regimen in this study was based on the specific condition that the patient was in the acute inflammatory phase. Additionally, the course of corticosteroid treatment was strictly controlled (≤ 4 weeks) to reduce long-term risks.

In conclusion, consensus on the etiology and standardized management of LMDF remains elusive. Clinically, LMDF overlaps with other facial inflammatory/granulomatous papular dermatoses, making histopathological examination indispensable for its diagnosis and differentiation from acne, sarcoidosis, and lymphomatoid papulosis. In this case, the patient achieved significant resolution of facial lesion and regrowth of bilateral eyebrows after receiving systemic prednisone and doxycycline. This not only validates the combination regimen’s efficacy in LMDF, but also highlights eyebrow alopecia as an atypical manifestation of the disease – emphasizing the need to explore underlying pathophysiological links to hair loss.

This study lacks direct evidence from the patient’s tissue, such as *P. acnes*-specific staining (e.g., Gram staining, PCR for P. acnes DNA) in eyebrow follicles and TGF-β1 immunohistochemistry in lesional skin. These tests could have verified the presence of *P. acnes* and the expression level of TGF-β1 in the eyebrow area, thereby providing stronger support for the proposed mechanism. Future studies with larger sample sizes and comprehensive pathological assessments are needed to clarify these mechanisms and optimize LMDF therapieutic strategies.

## Author contributions

**Writing – original draft:** Yuanting Su.

**Writing – review & editing:** Wei Cui.
